# Vaccinating The Invisibles, the COVID-19 vaccination of irregular migrants in Rome

**DOI:** 10.1093/eurpub/ckac131.521

**Published:** 2022-10-25

**Authors:** O Punzo, C Ferrara, S Pierconti

**Affiliations:** 1 CNEC, Istituto Superiore di Sanità, Rome, Italy; 2 INMP, Rome, Italy

## Abstract

**Background:**

The death toll of the COVID-19 pandemic has been hugely downsized by the advent of the currently available vaccines, at least in high-income countries. Nevertheless, there is a huge gap between countries and within countries with respect to vaccination access and coverage. In June 2021, several non-governmental non-profit organizations responded to a call issued by Regione Lazio to help organize and enrol for vaccination vulnerable people such as irregular migrants and migrants living in temporary or informal settlements. Through a huge effort, non-profit voluntary organizations Nonna Roma and Intersos enabled the vaccination of thousands of immigrants from different countries, age and residence status.

**Methods:**

We obtained a dataset regarding part of the people these NGOs facilitated in accessing the vaccination booking. We had information regarding age, sex, country of origin, nationality and type of document.

**Results:**

These populations were from different ethnic backgrounds, in majority males and below 50 years old on average. We found an association between the continent of origin and holding any document and between age and holding any document, both at a significant level (p < 0.05). Coming from Asia, as opposed to coming to any other continent, and being younger, i.e. under 25 years of age, were associated with holding any type of document.

**Conclusions:**

We reflect on the need of rethinking the services for this population, having in mind article 25 of the Universal Declaration of Human Rights, but also, more practically, that often these people work in our elderly homes as caregivers or as cleaners in our offices. Therefore, making it easier to access health services would be in any country’s interest, especially during a pandemic. Policy efforts directed at facilitating migrant access to health services would ultimately help create a safer community for both migrants and residents, for whom migrants often work as strategic employees.

**Key messages:**

• Irregular migrants have not been included in national vaccination plans everywhere, despite WHO and other entities recommendations.

• An integrated national plan for irregular migrants vaccination would be useful for protecting other vulnerable populations such as elderly.

